# Measurement properties of the Brazilian Portuguese version of the Lower-Extremity Motor Activity Log for chronic hemiparetic poststroke patients

**DOI:** 10.1055/s-0043-1767826

**Published:** 2023-05-09

**Authors:** Elaine Menezes-Oliveira, Marília Escudero Cecconi, Clarissa Barros de Oliveira, Milena Vegas, Sandra Regina Alouche, Ricardo Mario Arida, Gabriela da Silva Matuti

**Affiliations:** 1 Universidade Federal de São Paulo, Programa de Neurologia/Neurociências, São Paulo SP, Brazil.; 2 Associação de Assistência à Criança com Deficiência, Departamento de Fisioterapia de Adultos, São Paulo SP, Brazil.; 3 Universidade Cidade de São Paulo, Departamento de Fisioterapia, São Paulo SP, Brazil.; 4 Universidade Federal de São Paulo, Departamento de Fisiologia, São Paulo SP, Brazil.

**Keywords:** Stroke, Evaluation Study, Gait, Postural Balance, Acidente Vascular Cerebral, Estudo de Avaliação, Marcha, Equilíbrio Postural

## Abstract

**Background** Stroke is among the three leading causes of disability around the world, and it results in immediate difficulty in mobility and gait. There is a lack of instruments to evaluate what daily life is like for these individuals using their lower limbs in real-life environments (outside of the clinical environment).

**Objective** To perform the translation and cultural adaptation to Brazilian Portuguese of the Lower-Extremity Motor Activity Log (LE-MAL) and test its measurement properties in chronic poststroke individuals.

**Methods** The LE-MAL was translated into Brazilian Portuguese and adapted to the Brazilian culture. The comprehension and relevance of the final version were analyzed by a committee of specialists. The reliability, validity, and responsiveness of the LE-MAL/Brazil to detect changes after lower extremity constraint-induced movement therapy (LE-CIMT) and an intensive conventional therapy were tested.

**Results** The LE-MAL/Brazil showed excellent inter- and intrarater reliability, with an intraclass correlation coefficient and Cronbach alpha > 0.70, as well as standard error of measurement and smallest detectable change < 10% of the total instrument score when applied by the same evaluators.

**Conclusion** The responsiveness of the LE-MAL/Brazil to detect changes showed better results after LE-CIMT than after the intensive conventional therapy, with most of the correlations > 0.50.

## Resumo

**Antecedentes** O acidente vascular cerebral (AVC) é a terceira causa de deficiência ao redor do mundo, e resulta em dificuldades imediatas relativas à marcha e à mobilidade. Nota-se a ausência de instrumentos que avaliem o dia a dia desses indivíduos quanto ao uso dos membros inferiores em ambientes reais (fora do ambiente clínico).

**Objetivo** Traduzir e fazer a adaptação cultural para o português do Brasil da Lower-Extremity Motor Activity Log (LE-MAL), e testar suas propriedades psicométricas em indivíduos hemiparéticos crônicos pós-AVC.

**Métodos** A LE-MAL foi traduzida para o português do Brasil e adaptada para a cultura brasileira. A compreensão e a confiabilidade da versão final foram testadas por um comitê de especialistas. A confiabilidade, a validade e a responsividade em detectar mudanças após a terapia de contensão induzida para membros inferiores (TCI-MMII) e uma terapia convencional também foram testadas.

**Resultados** A LE-MAL/Brazil mostrou excelente confiabilidade intra e interavaliador, com coeficiente de correlação intraclasse e alfa de Cronbach > 0,70, bem como erro padrão da medida e mínima mudança detectável < 10% da pontuação total quando aplicada pelo mesmo avaliador.

**Conclusão** A responsividade da LE-MAL/Brazil em detectar mudanças após a TCI-MMII apresentou resultados melhores do que após a terapia convencional, com a maioria das correlações > 0,50.

## INTRODUCTION

Stroke is the third leading cause of disability around the world. 1 Most of the stroke survivors (approximately 2/3) will have immediate deficits on gait, and 30% of these individuals will not achieve the predicted distance on the Six-Minute Walk Test (6MWT) after 6 months of the onset of stroke. 2 3 4 Instruments were developed to measure and monitor gait evolution after a stroke; however, they assess the individuals' capability, such as gait speed, distance walked, and mobility and balance. 5 6 7 8 Still, it is important to evaluate the individual through aspects other than just capability, as preconized by International Classification of Functionality (ICF).

The Lower-Extremity Motor Activity Log (LE-MAL) is a scale developed to evaluate performance in real life situations through the activity domain of the ICF. 9 The LE-MAL contains 3 subscales: Assistance, Performance, and Confidence, and it evaluates 14 daily life tasks. 10 The reliability and validity of the original English version were tested among chronic poststroke patients, and since then it has been used as the main measure for lower extremity constraint-induced movement therapy (LE-CIMT). Additionally, the LE-MAL showed adequate reliability and validity for application in individuals with multiple sclerosis (MS). 10 11 12

For an adequate utilization of the clinical assessment instruments from one language to another, a cross-cultural adaptation process and analysis of measurement properties of the adapted version are required. 13 Recently, the Brazilian version of the LE-MAL (LE-MAL/Brazil) had its properties analyzed, such as reliability, validity, and floor and ceiling effects. 14

Therefore, the present study aimed to analyze certain measurement properties of the LE-MAL/Brazil for chronic hemiparetic poststroke patients. The instrument's responsiveness to two different intervention protocols was tested.

## METHODS

The Ethics Committee at Associação de Assistência à Criança Deficiente (AACD) approved the study (CAAE: 39743720.1.0000.0085, no. 4.420.927). Dr Edward Taub, the author of the original English version, authorized the study.

### Participants

In total, 83 participants were recruited from the Physiotherapy Department. The inclusion criteria were patients with a medical diagnosis of stroke, in the chronic phase of recovery (> 6 months), and with hemiparesis caused by stroke; the exclusion criteria were speech deficits that incapacitated the participant to understand and/or answer properly the evaluation scale and absence in the second or third moments of evaluation.

The participants were requested to be available to answer the questions of the scale at three different occasions (test, retest, and second evaluator).

### Procedures

All the procedures of the present study followed the guidelines proposed by Beaton et al. 13

First phase – translation and cultural adaptation of the instrument: two independent bilingual translators (1 lay translator and 1 physiotherapist specialized in neurological rehabilitation) translated the LE-MAL into Brazilian Portuguese. A synthesis between the two translated versions was created by comparing them with the original version, resulting in a single final version, which was adapted to the Brazilian culture.

This version was analyzed by a committee composed of thirty specialists involved in the neurological rehabilitation of adult patients. They assessed the comprehension and clinical relevance of each item, creating a new version with their suggestions. Two other independent translators performed a back translation (1 lay translator and 1 physiotherapist specialized in neurological rehabilitation), and this final version was evaluated and approved by the original authors.

Second Phase – analysis of measurement properties: the final and approved version of the LE-MAL/Brazil was applied to 83 hemiparetic poststroke participants. The instrument's reliability, validity, and responsiveness were evaluated.

The LE-MAL/Brazil was applied through a structured interview according to the original instructions. First, the participant was introduced to the instrument and given instructions on how to answer and score the questions. The time frame “last week” was adopted as the reference period for participants to answer about their need for assistance, performance, and confidence regarding 14 daily activities.

### Instruments

The LE-MAL is a questionnaire conducted by a therapist that evaluates the real use of the most affected lower extremity during 14 daily activities. It has three subscales: 1) functional performance during the task; 2) confidence while performing the task (fear of falling); and 3) need for and amount of assistance while performing the task (need for orthosis, gait aid, environment changes, and/or help from another person). Performance is measured through a Likert scale (0-10) of the self-perception of movement quality while performing the task (0 denotes “not able to do/did not realize”, and 10 denotes “normal movement”). Confidence is related to “how confident the patient was while performing the same task without fear of falling” (0 denotes “no confidence at all”, and 10 denotes “totally confident”). 10 11 12

Valid and more frequently-used instruments were selected to evaluate the gait, but once they measure motor capability they were considered concurrent instruments for the present study. The instrument chosen to evaluate performance was deemed convergent for the analysis.

The following instruments were deemed divergent: 6MWT, 15 16 17 the Ten-Meter Walk Test (10MWT), 17 18 and the Timed Up and Go Test (TUG) 6 7 for gait and mobility capability; and the Mini-Balance Evaluation Systems Test (Mini-BESTest) for balance. 19 20 21 22

As a convergent instrument, the Stroke Impact Scale (SIS), version 3.0, was selected; it detects permanent conditions caused by stroke. 23 For the present study, only the mobility domain was selected, which evaluates nine activities usually performed in real life, such as remaining standing without losing balance, transference from bed to wheelchair, and locomotion at home and outside of the house. The patient is asked to score their level of difficulty in completing each task in a scale ranging from 1 (unable to do task) to 5 (no perceived difficulty). The reference period is the previous two weeks. 23

### Measurement properties of the instrument

The reliability, validity, and responsiveness of the LE-MAL/Brazil were tested. The reliability was tested through inter- and intrarater analyses, ceiling/floor effect, standard error, and smallest detectable change (SDC). The construct validity was tested by comparing the LE-MAL/Brazil to concurrent and divergent instruments. The responsiveness was analyzed to detect changes after two interventions (effect size): LE-CIMT and an intensive conventional therapy and by comparing the LE-MAL/Brazil to concurrent and divergent instruments. 24 All the procedures were conducted following the recommendations proposed by the Consensus-Based Standards for the Selection of Health Measurement Instruments (COSMIN). 25

### Reliability

To analyze the reliability, the first contact with the participant was considered the first moment, when the scale was first applied. After two to three days, at the second moment, the same evaluator reapplied the scale. The third moment followed another interval of two to three days, when the second evaluator applied the scale for the first time.

Our a priori hypotheses were that interrater concordance would be high, ≥ 0.70, the standard error and the SDC would be ≤ 10% of the total score, and that the ceiling/floor effect of the sample would be ≤ 15%. 26 27

### Validity

Data were collected at the initial evaluations in both intervention groups, and data from the other evaluations were collected prospectively. 28

### Responsiveness

The responsiveness to detect changes after interventions was tested in two physiotherapy intervention protocols, the LE-CIMT and and intensive conventional therapy. Data were collected retrospectively at the initial (baseline) moment, the final moment (posttreatment), and at the 6-month follow-up evaluations of both interventions. 24

### Interventions

The LE-CIMT protocol was developed as a method to engage patients in order to transfer the gains made in the clinical setting to the real world, and the LE-MAL was first developed to better evaluate and monitor functional performance in real-life environments. It is for these reasons that we chose to test the responsiveness of this specific intervention. 11 However, the LE-MAL could be applied to any other intervention that proposes to improve balance and gait, so we tested its responsiveness in a conventional training of gait and balance.

Both interventions were conducted for 2.5 hours/day for 15 consecutive days of motor training; additionally, for the LE-CIMT group, 30 minutes were dedicated to the transfer package, as previously reported by Menezes-Oliveira et al (2021). 29

### Statistical analysis

Descriptive statistics and measures of frequency were used to analyze the sociodemographic and clinical data from 83 patients who completed the study.

To test the reliability, the internal consistency was analyzed using the Cronbach alpha, and the inter- and intrarater concordance, through the intraclass correlation coefficient (ICC). The ICC was considered as follows: < 0.50–weak concordance; between 0.50 and 0.70–moderate concordance; and > 0.70–strong concordance. The standard error was tested through the formula SD * √1 − ICC, and the SDC, through 1.96 * Standard Error * √2, and < 10% of the total score (1 point) was considered adequate. The ceiling/floor effect was tested through the partial frequency of minimum (zero) and maximum (ten) scores, and < 15% of the sample was considered adequate. 23 25 Additionally, our hypotheses were that the interrater concordance would be high, ≥ 0.70, the standard error and the SDC would be ≤ 10% of the total score, and that the ceiling/floor effect of the sample would be ≤15%. 26 27

Construct validity was tested through Pearson correlation for the LE-MAL/Brazil scores (assistance, performance, confidence, and total score) and the concurrent measures outcomes (Mini-BESTest, 10MWT, 6MWT, and TUG), adopting as a hypothesis < 0.70 as a moderate-to-weak correlation. For the convergent measure (SIS), the hypothesis of > 0.70 was adopted as a good correlation *.* Also, in the interrelation of the three subscales (assistance, performance, and confidence) and the total score through Pearson correlation > 0.70 was considered a good correlation.

The responsiveness to detect changes after LE-CIMT and the intensive conventional therapy was analyzed through the correlation of the LE-MAL/Brazil and the divergent tests of motor ability through the Pearson correlation *.* We expected that after both interventions motor ability would be aligned with the need for assistance, perception of performance, and confidence, so > 0.70 was considered an adequate correlation. 27 30

For responsiveness, the effect size was also calculated through the paired samples *t* -test of the G*Power 3.1.9.7 Heinrich-Heine-University software considering the mean and standard deviation values pre- and posttreatment, and the posttreatment and six-month follow-up values for the three subscales and the total score. The conventional effect sizes proposed by Cohen for the *t* -test are: 0.2 (small effect), 0.5 (moderate effect) and 0.8 (large effect). 31

The statistical analysis was conducted using the Statistical Package for the Social Sciences (SPSS Inc., Chicago, IL, US) software for Windows, version 13.0, and the level of significance adopted was α < 0.05.

## RESULTS

### Translation and cultural adaptation

The instrument was translated into Brazilian Portuguese, and the specialist committee made cultural adaptations before the form was semantically analyzed. In item 9 (“Getting in and out of the shower area”) on the subscale of Assistance (A2) and score 2, we changed “Used a transfer bench for tub” to “Used a bathing chair with a board for transfer”, because Brazilian houses do not usually have a tub. In item 13 (“Reaching into cabinets/closets – above shoulder level”) on subscale of Assistance (B3), the equipment suggested by the authors (“reacher”) was changed for “any type of external apparatus”.

These adaptations were presented and approved by the original authors, and the comprehension and clinical applicability of the 14 items of the back-translated version was analyzed by the specialist committee.

None of the items was scored ≤ 7 by more than 20% of the specialists. Nevertheless, item 5 (“Turning around when standing”) was scored ≤ 7 by 16% of the specialists in terms if comprehension, which made us accept the suggestion to include an additional description (“Changing direction when standing, a 180° turn). After including this description, there were no longer disagreements regarding the item, it could be clearly comprehended, and the suggestion was maintained.

### Measurement properties

To analyze the measurement properties of the LE-MAL/Brazil, 83 participants were recruited: 50 of them were part of the reliability and validity process, 20 of them participated in the responsiveness process as part of LE-CIMT group, and 19 participated in the responsiveness process as part of intensive conventional therapy group ( Table 1 ).

**Table 1:** Characterization data

Characterization data	Mean ± standard deviation
Age (years)	52 ± 16
Sex (%)	Female: 56; male: 44
Time since stroke (months)	35 ± 26
Assistance subscale (score)	8 ± 2
Performance subscale (score)	6 ± 2
Confidence subscale (score)	6 ± 2
Mini-Balance Evaluation Systems Test (score)	11 ± 5
Ten-meter walk test (m/s)	0.44 ± 0.30
Six-minute walk test (meters)	152.12 ± 113.49
Timed Up and Go (seconds)	40.29 ± 36.86
Stroke Impact Scale (score)	35 ± 8

The reliability of the LE-MAL/Brazil was tested through an analysis of the internal consistency and inter- and intrarater concordance ( Table 2 ), standard error of measurement, and the SDC ( Table 3 ).

**Table 2:** Internal consistency and inter- and intrarater concordance for the LE-MAL/Brazil

Subscales		Cronbachs alpha	ICC	95%CI	
				Inferior	Superior
Assistance	Interrater	0.95	0.91	0.85	0.95
	Intrarater	0.99	0.98	0.97	0.99
Performance	Interrater	0.91	0.84	0.73	0.91
	Intrarater	0.95	0.90	0.84	0.95
Confidence	Interrater	0.97	0.94	0.90	0.98
	Intrarater	0.96	0.92	0.86	0.95
Total score	Interrater	0.86	0.76	0.61	0.85
	Intrarater	0.92	0.84	0.74	0.90

Abbreviations: 95%CI, 95% confidence interval; ICC, intraclass correlation coefficient; LE-MAL/Brazil, Brazilian Portuguese version of the Lower-Extremity Motor Activity Log (LE-MAL).

**Table 3:** Standard error of measurement and smallest detectable change (SDC) for LE-MAL/Brazil

Subscales		Standard error of measurement	SDC
Assistance	Interrater	0.23	0.91
	Intrarater	0.05	0.20
Performance	Interrater	0.50	1.96
	Intrarater	0.32	1.25
Confidence	Interrater	0.19	0.74
	Intrarater	0.28	1.10
Total score	Interrater	0.21	0.58
	Intrarater	0.17	0.47

Abbreviation: LE-MAL/Brazil, Brazilian Portuguese version of the Lower-Extremity Motor Activity Log (LE-MAL).

The LE-MAL/Brazil was shown to have excellent inter- and intrarater reliability (Cronbach alpha and ICC >0.70) for the three subscales and the total score. The standard error of measurement and the SDC were adequate for the three subscales and the total score regarding intrarater data. The Performance subscale showed a value higher than that of our a priori hypothesis, in which the SDC would be ≤ 10% (up to 1 point) of the total score (considering that the maximum score for this scale is 10) regarding interrater application.

The LE-MAL/Brazil showed significant correlations with all the divergent instruments tested, except for the Confidence Subscale and the 10MWT. All the a priori hypotheses were confirmed ( Table 4 ), namely that all the correlations with the divergent instruments (Mini-BESTest, 10MWT, 6MWT, and TUG) would be < 0.70, thus showing a moderate-to-weak correlation. The correlation with the convergent instrument (SIS) was > 0.70 for Assistance, thus showing a strong correlation, and near to 0.70 for Performance, Confidence, and the total score.

**Table 4:** Content validity of LE-MAL/Brazil

Subscales	Convergent instrument	Divergent instruments			
	SIS	Mini-BESTest	10mWT	6mWT	TUG
Assistance	0.80**	0.57**	-0.30*	0.62**	-0.48**
Performance	0.65**	0.51**	-0.29*	0.54**	-0.41**
Confidence	0.68**	0.50**	-0.25	0.51**	-0.36**
Total score	0.67**	0.52**	-0.22	0.51**	-0.35**

Abbreviations: 10MWT, Ten-Meter Walk Test; 6MWT, Six-Minute Walk Test; LE-MAL/Brazil, Brazilian Portuguese version of the Lower-Extremity Motor Activity Log (LE-MAL); Mini-BESTest. Mini-Balance Evaluation Systems Test; SIS, Stroke Impact Scale; TUG, Timed Up and Go.

Notes: * *p* < 0.05; ** *p* < 0.01.

The construct validity of the LE-MAL/Brazil was considered strong, as it had a significant and strong interrelation for all the subscales (Assistance versus Performance: 0.75; Assistance versus Confidence: 0.71; Performance versus Assistance: 0.75; Performance versus Confidence: 0.94; Confidence versus Assistance: 0.71; and Confidence versus Performance: 0.94).

The responsiveness of the LE-MAL/Brazil to detect changes after LE-CIMT and the intensive conventional therapy was analyzed through its correlation with motor ability tests (divergent instruments). The correlation was shown to be moderate to weak ( Table 5 ), with better results in favor of the LE-CIMT protocol (correlations > 0.50).

**Table 5:** Responsiveness of the LE-MAL/Brazil to the LE-CIMT protocol and the intensive conventional therapy

Subscales		Mini-BESTest	10MWT	6mWT	TUG
Responsiveness to the LE-CIMT protocol	Assistance	0.56*	-0.59*	0.63**	-0.62**
	Performance	0.63**	-0.54*	0.56*	-0.63**
	Confidence	0.45	-0.46	0.47*	-0.55*
	Total score	0.57**	-0.55*	0.57*	-0.62*
Responsiveness to the intensive conventional therapy	Assistance	0.83**	-0.30	0.60*	-0.45
	Performance	0.55*	-0.08	0.22	-0.25
	Confidence	0.53*	-0.02	0.20	-0.22
	Total score	0.67**	-0.14	0.35	-0.32

Abbreviations: 10MWT, Ten-Meter Walk Test; 6MWT, Six-Minute Walk Test; LE_CIMT, lower extremity constraint-induced movement therapy; LE-MAL/Brazil, Brazilian Portuguese version of the Lower-Extremity Motor Activity Log (LE-MAL); Mini-BESTest, Mini-Balance Evaluation Systems Test; TUG, Timed Up and Go.

Notes: * *p* < 0.05; ** *p* < 0.01.

The posttreatment effect size for the LE-MAL/Brazil was of 0.34 for assistance, of 1.0 for performance, of 0.97 for confidence, and of 1.11 for the total score for LE-CIMT group; and of 0.23 for assistance, of 0.71 for performance, of 0.76 for confidence, and of 0.86 for the total score for the intensive conventional therapy group. On the 6-month follow-up, it was of 0.29 for assistance, of 0.57 for performance, of 0.63 for confidence, and of 0.39 for the total score for LE-CIMT group; and of 0.24 for assistance, of 0.51 for performance, of 0.56 for confidence, and of 0.41 for the total score for the intensive conventional therapy group.

In the analysis of the responsiveness of the LE-MAL/Brazil to detecting changes after LE-CIMT, we found a significant correlation with all the tested instruments, except for the Confidence subscale and the Mini-BESTest and the 10MWT, confirming the a priori hypotheses ( Table 5 ), as the correlation with the divergent instruments was between 0.30 and 0.70 (moderate correlations).

The responsiveness of the LE-MAL/Brazil to detect changes after the intensive conventional therapy presented a significant correlation with the Mini-BESTest in the three subscales and the total score, and with the 6MWT in the Assistance subscale. The a priori hypotheses were not completely confirmed ( Table 5 ), as most of the correlations were not significant; however, when a significant correlation was found, it was between 0.30 and 0.70, denoting a moderate correlation. The only strong correlation found was related to the Assistance subscale and the Mini-BESTest.

## DISCUSSION

The LE-MAL/Brazil presented excellent reliability, being easily reproduced in a consistent way when applied either by the same evaluator or by two different evaluators, yielding excellent internal consistency and concordance. The content validity showed a strong correlation, which suitably reflects the scale construct as a whole. The construct validity, when compared to the divergent instruments (Mini-BESTest, 10MWT, 6MWT, and TUG), presented a moderate-to-weak correlation. It confirmed the study hypothesis, thus reflecting the difference between the proposals of the LE-MAL/Brazil (to evaluate what the individual actually does in their habitual environment) and the divergent instruments, aimed to evaluate capacity (what the individual is able to do in a controlled/clinical environment).

A strong correlation was found between the LE-MAL/Brazil and the convergent instrument (SIS), which confirmed our a priori hypothesis, demonstrating that both instruments measure the same construct (performance). The responsiveness after LE-CIMT presented small changes after the intervention, showing a moderate correlation, whereas the responsiveness after the intensive conventional therapy showed a moderate correlation. The effect size was moderate to large for the LE-CIMT group, with a better responsiveness posttreatment, while it was small to moderate for the intensive conventional therapy group; this difference could be attributed to the fact that the LE-MAL was developed to monitor the changes after the LE-CIMT protocol. 10 11 32 However, it shows that the LE-MAL is sensitive to detect changes after a conventional therapy, specially using the total score to measure it.

According to our results, the LE-MAL/Brazil was shown to be a reliable, valid, and stable scale to measure and monitor changes in the functional performance of poststroke patients regarding everyday activities involving the use of the lower limbs.

The LE-MAL has been used as a fundamental part of the process of monitoring and following poststroke individuals when it comes to the performance of everyday activities using the lower limbs. 10 29 Recently, the scale had its measurement properties tested in its original version for individuals with MS 11 and stroke, 14 and presented good results regarding validity, reliability, and stability.

In the present study, we obtained similar scores for internal consistency when compared to the study 11 on MS patients, with values between 0.95 and 0.99, and 0.93 and 0.95 respectively, although our internal consistency was higher than that of the Brazilian study 14 on stroke patients, with values between 0.80 and 0.86. In this regard, the LE-MAL is a scale whose results are easily reproduced when applied more than once, and, in the case of the MS study, 11 by a single evaluator at different moments; our study expanded it to be used by different evaluators and at different moments. 11 Our internal consistency score for different evaluators obtained variations between 0.91 and 0.97.

Regarding the agreement values, we observed results similar to those of the MS study 11 : the ICC values ranged from 0.94 to 0.97. Our correlation values ranged from 0.90 to 0.98, moreover including different evaluators with scores between 0.84 and 0.94 for subscales. In the study 14 with stroke patients, the ICC values ranged from 0.51 to 0.94. This important difference between our results and those of the other Brazilian version could be justified due to differences in the sample size.

Comparing the validity results, MS patients presented values ranging from 0.56 to 0.79, since instruments deemed convergent, which are commonly used in the evaluation of MS, were selected. 11 Therefore, they presented values higher than 0.56, thus demonstrating a moderate-to-strong correlation. However, in all cases the LE-MAL stands out, more directly reflecting the performance of these individuals but showing similar correlation values between 0.53 and 0.83 (confidence versus Mini-BESTest: 0.53; and assistance versus Mini-BESTest: 0.83). The previously published Brazilian version of the LE-MAL 14 analyzed the validity with the Fugl-Meyer Assessment of Lower Extremity as a convergent instrument, and a correlation of 0.55 was found. In the present study, convergent and divergent instruments were analyzed. In the analysis of the convergent instrument (SIS), we found a good correlation with the LE-MAL/Brazil (assistance versus SIS: 0.80; performance versus SIS: 0.65; confidence versus SIS: 0.68; and SIS versus total score: 0.67). Patients with stroke usually present a good score in scales of capacity, demonstrating their physical ability to perform tasks, but this capacity is frequently not transferred to the real-life environment, which suggests that in spite of being physically able to perform the task, they often do not perform them on a day-to-day basis. 33

Currently, the literature demonstrates a scarcity of instruments to assess the performance of poststroke individuals in daily tasks involving the lower limbs. This scarcity is even more remarkable when searching for instruments that have already been translated and analyzed regarding the Brazilian population. Our results demonstrate that the LE-MAL/Brazil can be a viable instrument to meet this demand in this population.

In conclusion, the LE-MAL/Brazil was shown to be understandable, with relevant clinical applicability, excellent reliability (when applied by the same or by different evaluators), with adequate standard error of measurement and SDC.

The instrument demonstrated validity to evaluate the need for assistance, the self-perception of performance, and the confidence of chronic poststroke individuals when performing daily activities. It showed moderate responsiveness to detect changes after two distinct interventions, with better sensitivity when applied to monitor the results of LE-CIMT.
